# Transcriptomic Analysis of Gibberellin- and Paclobutrazol-Treated Rice Seedlings under Submergence

**DOI:** 10.3390/ijms18102225

**Published:** 2017-10-24

**Authors:** Jing Xiang, Hui Wu, Yuping Zhang, Yikai Zhang, Yifeng Wang, Zhiyong Li, Haiyan Lin, Huizhe Chen, Jian Zhang, Defeng Zhu

**Affiliations:** 1 State Key Lab of Rice Biology, China National Rice Research Institute, Hangzhou 311400, China; xiangjing_823@163.com (J.X.); wuhuiscience@163.com (H.W.); cnrrizyp@163.com (Y.Z.); Yikaizhang168@163.com (Y.Z.); wangyifeng@caas.cn (Y.W.); lzhy1418@163.com (Z.L.); 2 Yuan LongPing High-TechAgriculture Co., Ltd., Changsha 410001, China; linhaiyan@188.com

**Keywords:** rice (*Oryza sativa* L.), submergence, transcriptome, gibberellic acid, paclobutrazol

## Abstract

Submergence stress is a limiting factor for rice growing in rainfed lowland areas of the world. It is known that the phytohormone gibberellin (GA) has negative effects on submergence tolerance in rice, while its inhibitor paclobutrazol (PB) does the opposite. However, the physiological and molecular basis underlying the GA- and PB-regulated submergence response remains largely unknown. In this study, we reveal that PB could significantly enhance rice seedling survival by retaining a higher level of chlorophyll content and alcohol dehydrogenase activity, and decelerating the consumption of non-structure carbohydrate when compared with the control and GA-treated samples. Further transcriptomic analysis identified 3936 differentially expressed genes (DEGs) among the GA- and PB-treated samples and control, which are extensively involved in the submergence and other abiotic stress responses, phytohormone biosynthesis and signaling, photosynthesis, and nutrient metabolism. The results suggested that PB enhances rice survival under submergence through maintaining the photosynthesis capacity and reducing nutrient metabolism. Taken together, the current study provided new insight into the mechanism of phytohormone-regulated submergence response in rice.

## 1. Introduction

Rice (*Oryza sativa* L.) is a semi-aquatic plant that is well adapted to a partially flooded environment, but excess water caused by submergence or waterlogging hampers rice growth and survival, which could eventually lead to drastic yield losses [1,2]. It was estimated that 16% of the lowland rice in the world, and over 22 million hectares of Asian rainfed rice, are adversely affected by submergence stress [3,4]. Submergence severely restricts the gas exchange of O_2_ and CO_2_ between rice tissues and the atmosphere, inhibits aerobic respiration and photosynthesis, and accelerates energy reserve consumption, resulting in stunted growth or death [1,5,6]. In addition to the O_2_ deficiency, submergence could also affect plant growth by producing toxic substances through a reduction of the redox potential and increasing susceptibility to diseases [6]. In response to submergence, different rice ecotypes have developed different strategies to survive under stress. In some areas of Asia, submergence occurs very rapidly and lasts for months. The deepwater rice grown there takes a low-O_2_ escape syndrome (LOES) strategy, in which gibberellin (GA)-mediated internode elongation was promptly activated, enabling the plants to outgrow flood water for air exchange. Though LOES strategy works well for deepwater rice, extensive shoot elongation is not desirable for lowland cultivars under flash flooding conditions, because an elongated shoot usually leads to lodging and higher susceptibility to pathogens and pests after the water recedes [7]. In contrast to LOES, irrigated rice varieties withstand the submergence through a low-O_2_ quiescence syndrome (LOQS) strategy, which is characterized by a reduction of carbohydrate consumption, chlorophyll degradation, and growth elongation during submergence. A low metabolism level in the quiescence status saves energy to ensure rapid plant re-growth when the flood recedes [8,9,10]. 

So far, a series of agronomic techniques have been developed to improve the rice submergence tolerance. For example, proper nursery management of the seedlings with optimized fertilizer recipe, seed density, and transplanting time was found to be effective in improving rice yield under submergence [11]. Better submergence tolerance was also achieved by the application of growth regulators such as post-flood nitrogen, phytohormones, or hormone inhibitors [4,7,12]. It has been shown that submergence responses in rice are orchestrated by the interplay of various phytohormones, particularly ethylene, gibberellins (GA), and abscisic acid (ABA). For deepwater rice, which take a LOES strategy in submergence, anaerobic stress promotes gaseous ethylene biosynthesis and entrapment in rice tissues, which substantially repress the ABA level by inhibiting ABA production as well as ABA degradation [13,14,15]. The ABA decline consequently increases the GA sensitivity and/or production, resulting in the activation of a range of acclimation responses such as shoot elongation, adventitious root formation, and carbohydrate metabolism [16,17,18]. In this process, *SNORKEL1* (*SK1*) and *SNORKEL2* (*SK2*) containing an *APETALA2/Ethylene Response Factor* (*AP2/ERF*) domain are two of the most important regulators. Signaling by *SK* genes may be directly or indirectly connected to GA biosynthesis, which then induces internode elongation [19]. Conversely, rice quiescence during flash flooding was associated with a reduced level or responsiveness to GA [20]. From submergence-tolerant cultivar FR13A, Xu et al. (2006) identified a major locus controlling rice quiescence under submergence stress, *Sub1* [10]. The *Sub1* locus is comprised of a cluster of two or three tandem-repeated VII *ERF* genes, named *Sub1A*, *Sub1B*, and *Sub1C*. In contrast to the invariable presence of *Sub1B* and *Sub1C* in all *O. sativa* accessions, Sub1A only exists in submergence-tolerant varieties and could confer enhanced tolerance to submergence-intolerant plants, indicating that *Sub1A* is a primary determinant of submergence tolerance [10]. *Sub1A* expression is induced by the accumulation of ethylene under submergence, and its overexpression lines displayed typical GA-insensitive phenotypes. In support of this phenomenon, *Sub1A* was found to increase the accumulation of the GA signaling repressors Slender Rice-1 (SLR1) and SLR1 Like-1 (SLRL1), and hence to block the GA signaling pathway under submerged conditions [21]. In addition to the quiescence status during submergence, Sub1A may also participate in other post-submergence processes, such as oxidative stress response, leaf senescence, and floral initiation [22,23,24]. 

Although the submergence response, either in an LOES or LOQS manner, starts with ethylene accumulation, it seems that GA is more likely to be directly involved in submergence tolerance, whereas ethylene serves as an intermediate component that conveys the submergence signals to downstream factors affecting GA sensitivity or biosynthesis [25]. Dubois et al. (2011) investigated the role of GAs and ethylene in submergence response in two intolerant varieties, and found that the shoot elongation of variety Senia relied on the GA content level, but not on ethylene, indicating the existence of a GA-dependent, ethylene-independent pathway [26]. Given that the quiescence status of rice is essentially obtained by blocking the GA signaling and/or decreasing GA content, application of GA biosynthesis inhibitors, such as paclobutrazol (PB), is believed to be an alternative way to enhance rice submergence tolerance, especially in rice varieties without the *Sub1A* gene. Indeed, this hypothesis has been supported by numerous studies that showed that application of PB significantly increased the survival rate of rice by impacting several physiological aspects including shoot elongation, gas exchange, and photosynthesis [4,7,26,27,28]. Despite the fact that the key roles of GA in submergence have been widely accepted, the regulatory gene network and mechanism underlying the GA-mediated submergence response remain largely unknown. Here, we report a comprehensive transcriptomic analysis of the effect of GA and PB treatments on rice variety IR64, which takes a LOQS strategy in response to submergence. We aimed to find candidate regulatory genes and networks during the GA- or PB-regulated submergence response, particularly in submergence-intolerant varieties, which represents the majority of cultivars in China, so as to provide valuable clues for the identification of submergence-tolerant genes for genetic improvement. The outcome of this study is anticipated to enhance our understanding of the molecular basis of GA and its inhibitor in rice submergence tolerance.

## 2. Results

### 2.1. PB (Paclobutrazol) Enhanced the Survival Rate of IR64 under Submergence Stress

**Figure 1:**
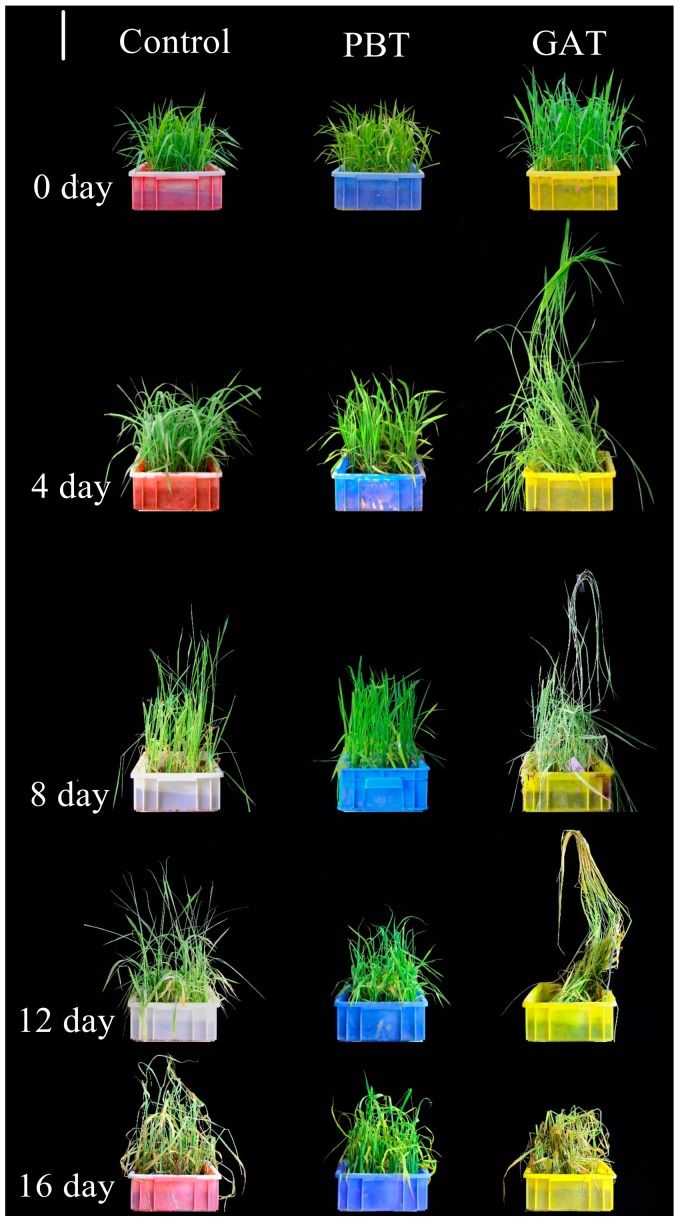
Phenotype of control, GAT (Gibberellin-treated) and PBT (paclobutrazol-treated) seedlings during submergence stress. The three columns show the control, GAT, and PBT seedlings, respectively. The five rows show seedlings that were submerged for 0, 4, 8, 12, and 16 days. Scale bar = 15 cm.

**Figure 2:**
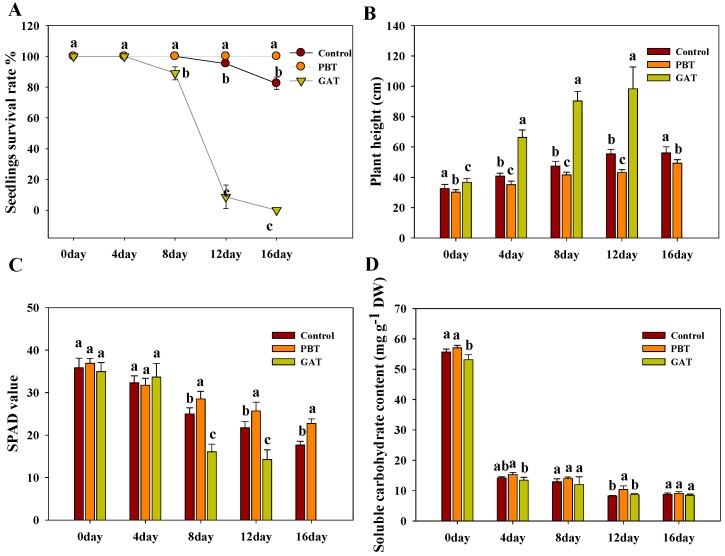

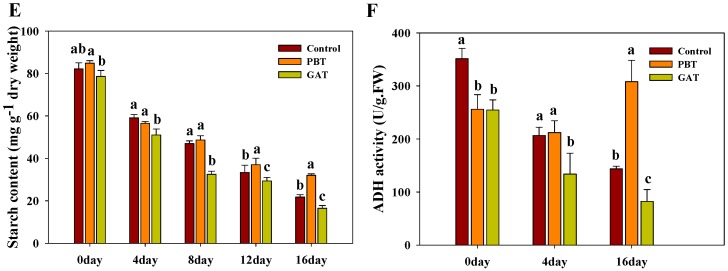
Physiological assay of control, GAT, and PBT seedlings under submergence. (**A**) Seedling survival rate of control, GAT, and PBT seedlings after submergence for 0, 4, 8, 12, and 16 days; (**B**) plant height of control, GAT, and PBT seedlings after submergence for 0, 4, 8, 12, and 16 days; (**C**) SPAD value of control, GAT, and PBT seedlings after submergence for 0, 4, 8, 12, and 16 days; (**D**) soluble carbohydrate content of control, GAT, and PBT seedlings after submergence for 0, 4, 8, 12 and 16 days; (**E**) starch content of control, GAT, and PBT seedlings after submergence for 0, 4, 8, 12 and 16 days; (**F**) ADH activity of control, GAT, and PBT seedlings after submergence for 0, 4 and 16 days. Values are the means ± SD (standard deviation) of 10 plants as 10 replicates for plant height and SPAD (soil and plant analyzer development) value, and four pots of plants as four replicates for NSC (non structural carbohydrate), and three pots of plants as three replicates for survival rate and ADH (alcohol dehydrogenase) activity. Bars with different letters indicate significant differences in samples among groups at the same time point at *p* < 0.05 (Duncan’s test).

Prior to the submergence, we conducted quantitative RT-PCR to check the transcriptional level of a GA-responsive marker gene *RGA1* (*LOC_Os05g26890*) in GA-Treated (GAT), PB-Treated (PBT), and methanol solvent-treated (control) samples [29]. The high level of *RGA1* in GAT and suppressed level in PBT confirmed that the chemical treatments were valid (Figure S1). Physiological properties of the samples were examined at 4, 8, 12 and 16 days after the stress. Firstly, the plant survival rate was tested to evaluate the overall performance of the GAT, PBT, and control in the tolerance of submergence. Because IR64 is a submergence-intolerant variety containing no *Sub1A* locus, control plants started to die at 12 days, and the final survival rate after 16 days was about 82.4%. Application of GA accelerated plant death as 11% had died within 8 days, and no GA plants could survive for 16 days. In contrast, PB significantly enhanced the plants’ performance during submergence, as we observed that 100% of the PB plants survived even after 16 days of stress (Figure 1 and Figure 2A). Submergence greatly induced the shoot elongation of IR64, but the extent of elongation varied significantly between the tested treatments. As shown in Figure 2B, the height of control plants increased from 32.5 cm at 0 to 56.1 cm after 16 days, accounting for an increment of 72.6%. Exogenous GA apparently promoted the submergence-induced elongation, with a height increase of 167.7% in 12 days (data at 16 day are not available due to the death of GA plants at 12 days), whereas PB plants only increased 63.0% in height in 16 days, indicating a predominant role of GA level in the shoot elongation under submergence (Figure 2B). Employing a SPAD (Soil and Plant Analyzer Development) system [30], we further examined the chlorophyll content of the plants. The results clearly showed that the SPAD value decreased with the time of submergence treatment for GA, PB, and the control. However, application of GA accelerated the chlorophyll degradation, which gave the plants a relatively lower chlorophyll level compared with the control. Meanwhile, application of PB, an inhibitor of GA, had the opposite effect on chlorophyll content (Figure 2C).

Due to the blocked photosynthesis, non-structure carbohydrates such as soluble carbohydrates and starch are consumed to maintain the basic metabolism of plants under water. As a direct source of energy, soluble carbohydrate was rapidly depleted in the first four days of submergence, and reached the same level for GA, PB, and the control after eight days (Figure 2D). We also observed a rapid consumption of starch for the three samples. It seemed that GA accelerated the starch depletion during submergence, possibly due to the robust metabolism activated by GA. Meanwhile, the starch content in PB was significantly higher than the other two samples after 12 days (Figure 2E). 

Alcohol dehydrogenase (ADH) activity is critical for rice to survive under anoxic conditions [31]. We observed that the ADH activities of GAT and PBT were significantly lower than the control at 0 day. However, the ADH activity of GAT and control dramatically decreased during submergence stress, while the ADH activity of PBT displayed the opposite tendency. Finally, PBT had a significantly higher ADH activity than GAT and the control at 16 days (Figure 2F).

### 2.2. Transcriptomic Analysis of Rice in Response to GA (Gibberellin) and PB under Submergence

**Figure 3:**
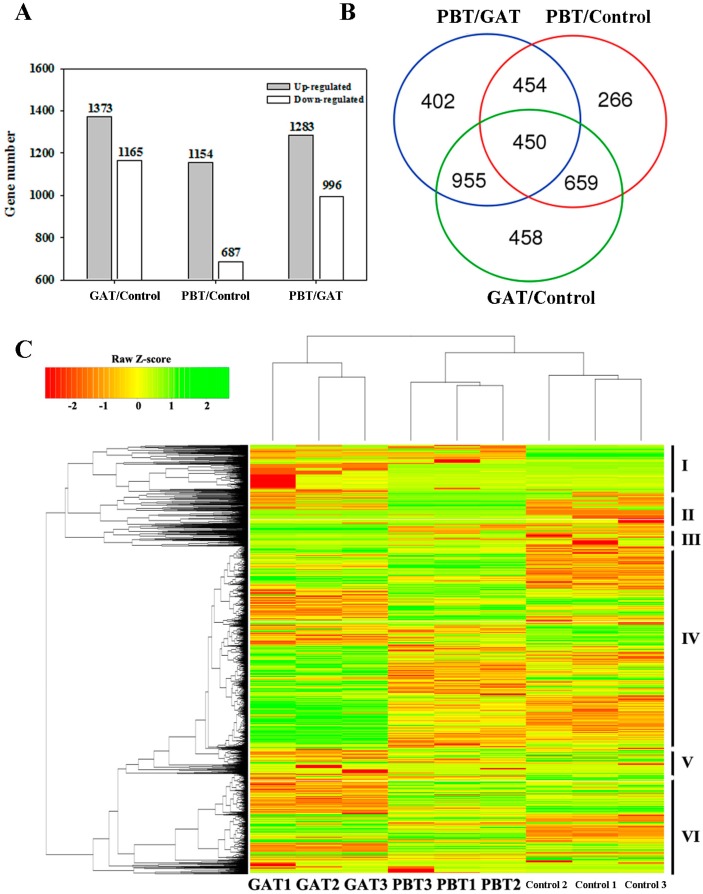
(**A**) Number of DEGs in different comparisons; (**B**) Venn diagram showing the commonly identified DEGs (differentially expressed genes) in different comparisons; (**C**) hierarchical clustering analysis of the DEGs (differentially expressed genes) among control, GAT, and PBT. The color bar on the left represents the log2 FPKM values. Red, yellow, and green indicate low, medium, and high FPKM (Fragments Per Kilobase of exon model per Million mapped reads) values, respectively.

We Illumina sequenced the cDNA libraries deriving from the seedlings of control, GAT, and PBT at 4 days, with biological triplicates. As a result, we obtained 43.8, 44.1, and 42.7 million clean reads in total, containing 6.6, 6.6, and 6.4 Gb high quality bases (Q30% > 90.1) for control, GAT, and PBT, respectively (Table S1). More than 90% of the reads could be mapped into the rice genome, including over 56.3% that were uniquely mapped, suggesting the successful performance of RNA sequencing in this study (Table S2). By using the FPKM (Fragments Per Kilobase of exon model per Million mapped reads) to quantify the transcription abundance, a total of 3936 DEGs were identified in at least one of the three comparisons, namely GAT/CK, PBT/CK, and PBT/GAT (|log2foldchange|≥1, *p* < 0.05) (Table S3). By searching against the plant transcription factor (TF) database (http://planttfdb.cbi.pku.edu.cn/) [32], we found that 301 of the DEGs are TFs belonging to *ERF* (*Ethylene Responsive Factor*), *WRKY*, *bHLH* (*basic Helix-Loop-Helix*), *bZIP* (*basic region/leucine Zipper*), and some other TF families (Table S3). In addition, 46 of the DEGs are defined as epi-genetic controlling genes in the Chromatin database (http://www.chromdb.org/) [33], which are extensively involved in chromatin remodeling, histone demethylation, and so on, suggesting the important roles of epigenetic regulation in response to submergence (Table S3). For the three comparisons, we identified 2538 (1373 upregulated and 1165 downregulated), 1841 (1154 upregulated and 687 downregulated) and 2279 (1283 upregulated and 996 downregulated) DEGs in GAT/Control, PBT/Control, and PBT/GAT, respectively (Figure 3A). While 1109 DEGs were identified in both GAT/Control and PBT/Control, and 1405 in both GAT/Control and PBT/GAT, 904 DEGs overlapped in PBT/GAT and PBT/Control. Moreover, we found that 450 DEGs were commonly present in all three comparisons (Figure 3B). Based on the transcription abundance, a hierarchical clustering analysis divided the DEGs into six major clades, indicating their divergent roles in submergence tolerance (Figure 3C). For example, DEGs in Clade I had a lower expression level in GAT, while the expression increased in the control and reached the maximum level in PB. Considering the best performance of PBT in submergence tolerance, such expression pattern indicated the positive roles of Clade I members in submergence tolerance. Meanwhile, DEGs in Clade II are proposed to be negative regulators because their expression levels are significantly higher in GAT compared with PBT and the control. In addition, the co-expression pattern of the DEGs in each clade implied their involvement in the same or similar pathways of submergence response.

### 2.3. GO (Gene Ontology) and KEGG (Kyoto Encyclopedia of Genes and Genomes) Enrichment Analysis of DEGs

**Figure 4:**
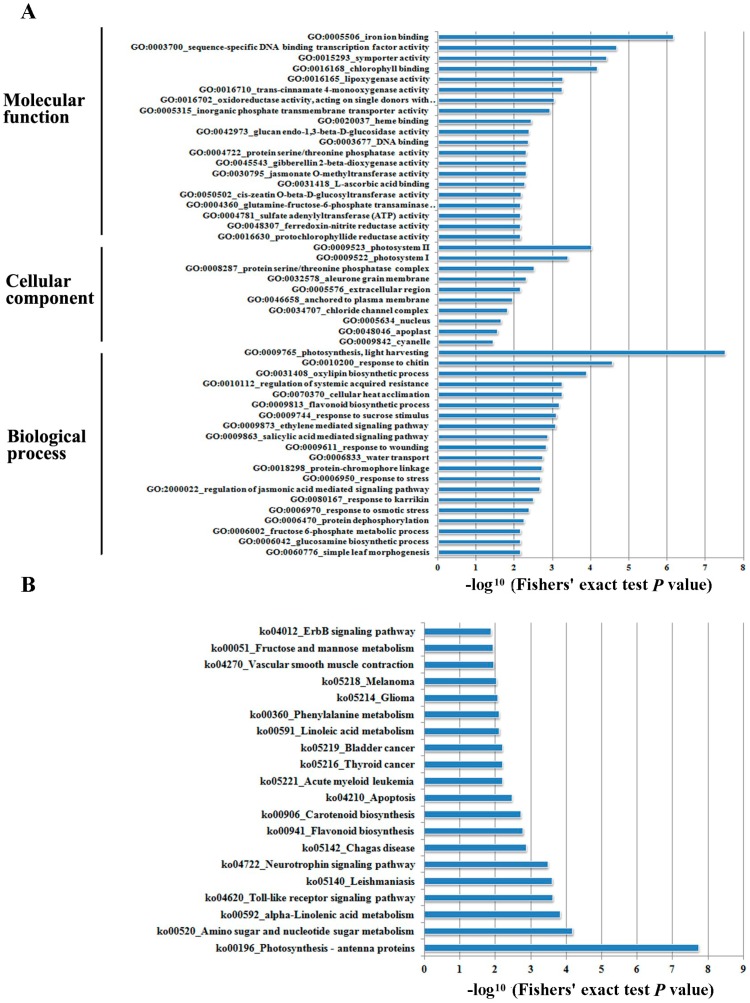
Enrichment analysis of DEGs based on the Gene Ontology (**A**) and KEGG pathway (**B**).

For a better understanding of DEG functions, GO (gene ontology) classification enrichment assay was performed on the 3936 DEGs in terms of biological process, cellular components, and molecular function (Figure 4A). From the “biological process” perspective, the DEGs were enriched in categories such as “regulation of transcription”, “defense response”, “stress response”, and “proteolysis” (*p* < 0.05). In particular, we found that DEGs were enriched in the “ethylene-mediated signaling pathway”, implying the critical role of ethylene in the submergence response. Similarly, DEGs were also commonly overrepresented in the “integral to membrane”, “plasma membrane”, “nucleus”, and “cytoplasm” categories when the “cellular component” is considered. In terms of “molecular function”, we found that GO enrichment occurred in the categories of “ATP binding”, “Protein Serine/Threonine kinase activity”, “receptor activity” and “DNA binding”.

Pathway-based enrichment analysis facilitates further elucidation of the biological functions of DEGs. In the current study, we conducted pathway-based analysis for the DEGs using the KEGG pathway database [34]. As a result, 483 genes were enriched in 43 biological pathways (Figure 4B). “Photosynthesis—antenna proteins” is the most significantly enriched pathway, as 10 out of the 15 member genes were present. Followed over-presented pathways include “amino sugar and nucleotide sugar metabolism”, “α-Linolenic acid metabolism”, “flavonoid biosynthesis”, “carotenoid biosynthesis”, “apoptosis”, “fructose and mannose metabolism”, and “MAPK signaling pathway”.

### 2.4. qRT-PCR Validation of DEGs

**Figure 5:**
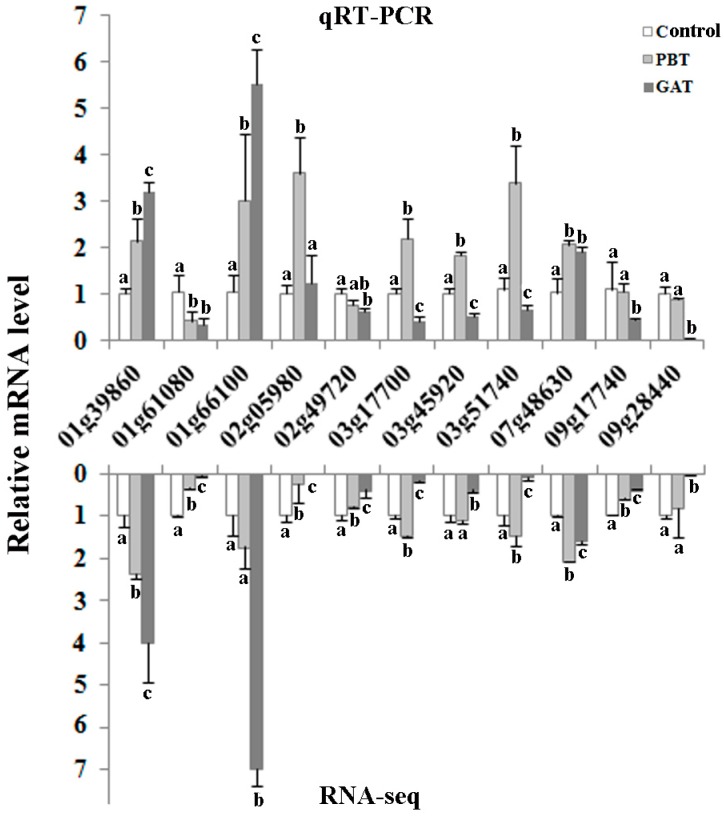
qRT-PCR validation of the DEGs in RNA-seq results. All values are based on three technical repeats and presented as means ± SD. Different characters indicate a statistically significant difference at *p* < 0.05 by *t*-test.

To validate the Illumina sequencing data and the expression patterns of the DEGs revealed by RNA-Seq, qRT-PCR was performed to examine the expression patterns of 11 DEGs (Figure 5). qRT-PCR results showed that the expression pattern of the majority of the DEGs exhibited same correlations with the RNA-seq data, though the regulation level may vary from genes. In particular, important submergence regulator genes, such as *EATB* (*ERF protein associated with tillering and panicle branching*) (*LOC_Os09g28440*) and *MPK3* (*LOC_Os03g17700*), showed exactly the same pattern as revealed by RNA-seq, suggesting the high reliability of the transcriptomic data in the current study.

## 3. Discussion

### 3.1. PB (Paclobutrazol) Decelerates the Physiological Activities of IR64 under Submergence

The low-O_2_ quiescence syndrome strategy is known as a key mechanism for rice to survive short periods of submergence [1]. As a key activator of many physiological processes, high GA content is not favored for rice recovery after submergence due to its acceleration effects on metabolism and energy depletion. Studies on rice submergence-tolerant varieties with the *Sub1A* locus demonstrated that a quiescence status is essentially achieved through blocking the signaling and/or biosynthesis pathways of GA. In recognition of this, application of exogenous GA inhibitors PB was believed to be an alternative means to enhance submergence tolerance [4,27]. Consistent with previous reports, the current study revealed a significantly positive impact of exogenous PB on rice submergence tolerance, while GA imposed opposite effects (Figure 1 and Figure 2A). Rice survival after submergence was found to be strongly dependent on non-structural carbohydrate reserves remaining in the shoot after submergence [4]. We also detected obvious retention of non-structure carbohydrates in the PB-treated plants, which may be attributed to the energy saved from reduced shoot elongation. Meanwhile, the repressed chlorophyll degradation in PBT may also help plants to maintain better photosynthesis capacity than in GAT and the control (Figure 2B–E). To support the survival of plants under water, soluble sugar, which is the direct source of energy, was rapidly consumed and depleted, decreasing to the same low level as in GAT, PBT, and CK (*p* < 0.05), though the PB-treated plants stored more pre-submergence soluble sugar (Figure 2D). Other sources such as starch are also used as energy supply through fermentative metabolism.

### 3.2. DEGs Related to Submergence and Other Abiotic Stress Responses

Although IR64 is a submergence-intolerant variety containing no *Sub1A* gene, we found a couple of other important submergence regulators are differentially expressed in response to the application of GA and PB. One well-documented example is *CIPK15* (*Calcineurin B-like interacting protein kinase 15*) (*LOC_Os11g02240*) [35]. CIPK15 is a plant-specific Ser-Thr protein kinase containing a conserved NAF (Asn-Ala-Phe) domain, which is necessary and sufficient for the interaction with calcineurin B-like proteins. *cipk15* mutants exhibited hypersensitivity to anaerobic germination under water. CIPK15 senses O_2_-deficiency signals, and works on the upstream of *SnRK1A*-*MybS1*-*Ramy3D* sugar signaling cascade to promote the starch degradation for rice growth under floodwater [35]. Research has also suggested that Sub1A and CIPK15 might play an antagonistic role in terms of carbohydrate consumption, and Sub1A could suppress the CIPK15 pathway under O_2_ deprivation [36]. In this study, we found that *CIPK15* was significantly upregulated by GA (fold change = 2.68, *p* < 0.05), whereas PB treatment kept the *CIPK15* expression at the same level as the control (*p* > 0.05), suggesting that PB-mediated submergence tolerance is, at least partially, ascribed to the retention of *CIPK15* expression. Another submergence-related DEG is *MPK3* (*LOC_Os03g17700*), belonging to the Mitogen-activated protein kinase gene family. In the background of Swarna with the presence of *Sub1A*, MPK3 is activated by submergence, and regulates the transcript accumulation of GA signaling genes *SLR1* and *SLRL1*. The MPK3-Ox Swarna-Sub1 line showed restricted shoot length elongation post-submergence, whereas the MPK3-silenced Swarna-Sub1 lines showed a chronic increase in shoot length post submergence, suggesting the positive role of MPK3 in submergence tolerance. Moreover, MPK3 physically interacts with and phosphorylates SUB1A, which could in turn bind to the *MPK3* promoter and regulate its expression in a positive regulatory loop during submergence stress signaling [37]. In our transcriptome data, we found a dramatic downregulation of MPK3 in the GAT/Control (fold change = 2.68, *p* < 0.05), but a similar expression level in the PBT/Control (*p* > 0.05). Given the positive role of *MPK3* in submergence tolerance, the downregulation pattern of it in GAT is consistent with the lower survival rate in GAT. Meanwhile, PB could maintain MPK3 at a relatively high level and hence enhance plant survival under water. In addition to the important submergence regulators, we found a number of abiotic resistant genes in our DEG list, including *ALDH2a* (*Aldehyde Dehydrogenase*) (LOC_Os02g49720), which is involved in submergence response [38], *OsbHLH1* (*basic helix-loop-helix*) (*LOC_Os07g43530*), which is implicated in cold stress signaling [39], and *MSR2* (*Multi-Stress-Responsive gene 2*) (*LOC_Os01g72530*), which participates in drought and stress resistance [40].

### 3.3. DEGs Involved in Phytohormone Biosynthesis and Signaling

Submergence tolerance is a complex event orchestrated by various phytohormones. As an inhibitor of GA, PB is expected to block the GA biosynthesis pathway. In the current study, we found the transcriptional level of *SD-1* (*Semi dwarfing 1*) (*LOC_Os01g66100*) in GAT was significantly higher than the control and PBT. Given that *SD-1* encodes a gibberellin 20-oxidase, which is a key enzyme in the conversion of bioactive GA forms, the suggestion is that *SD-1* might be a key node of the GA biosynthesis pathway in PB-mediated suppression. As a major regulator of submergence responses in rice, ethylene rapidly accumulates during stress and triggers a range of acclimation responses. ERF is a subgroup of the APETALA2 (AP2)/ERF family transcription factors that have been shown to bind specifically to the GCC box (AGCCGCC) of the ethylene-responsive element (ERE) [41]. Previous studies have linked several ERFs, such as the most famous Sub1A, with submergence tolerance [10]. Interestingly, among the 163 ERF TFs in the rice genome [32], 22 and eight ERFs were differentially expressed in GAT/Control and PBT/Control, respectively. In GAT/PBT, we found that the expression of *EATB* (*ERF protein associated with tillering and panicle branching*) (*LOC_Os09g28440*) was significantly repressed by GA (*p* < 0.05). It has been shown that *EATB* is ethylene responsive, and it suppresses the transcription of gibberellin biosynthetic gene *ent*-kaurene synthase A, which finally restricts the internode elongation [42]. The repressed expression of *EATB* by GA implies a feedback loop regulation in the ethylene–GA interaction. Meanwhile, the application of PB kept the *EATB* at a comparable level to the control (*p* > 0.05), which may help plants maintain a relatively low level of endogenous GA, and enhance survival under submergence. We also found that a number of other ethylene biosynthesis or signaling genes displayed the downregulation pattern in GAT/Control and remained unchanged in PBT/Control. These include *OsACO7* (*aminocyclopropane-1-carboxylate oxidase*) (*LOC_Os01g39860*) [43], *OsACS1* (*aminocyclopropane-1-carboxylate synthase*) (*LOC_Os03g51740*) [44], and *OsEIL2* (*Ethylene Insensitive 3-Like*) (*LOC_Os07g48630*) [45]. In addition to the GA- and ethylene-related genes, many other phytohormone-related genes were found to be differentially expressed in this study. These include ABA-related genes *OsNCED3* (*9-cis-epoxycarotenoid dioxygenase*) (*LOC_Os03g44380*), *DSM2* (*β-Carotene Hydroxylase*) (*LOC_Os03g03370*) [46,47]; BR (brassinosteroid)-related genes *DWARF4* (*LOC_Os03g12660*), *SERK2* (*somatic embryogenesis receptor kinase*) (*LOC_Os04g38480*) [48,49]; and auxin-related genes *ARF8* (*auxin response factor*) (*LOC_Os02g41800*), *OsNPR1* (*Nonexpressor of PR genes 1*) (*LOC_Os01g09800*) [50,51].

### 3.4. Photosynthesis and Nutrient Metabolism-Related DEGs

It was suggested that rice seedling survival is strongly associated with NSC (non-structural carbohydrate) content post-submergence, which is jointly determined by the photosynthesis rate and energy consumption rate during submergence [4]. In plants such as rice and other organisms, photosynthesis is a process that converts light energy into chemical energy in the form of carbohydrate molecules, which could later be released to fuel necessary biological activities. Under submergence, in addition to the blocked light accessibility to plant photosynthesis organs and stress-induced chlorophyll degradation, maximum quantum efficiency, which is a key parameter determining the function and stability of PSII (Fv/Fm), was also found to be drastically declined [27,52,53]. Meanwhile, several previous studies, along with the current study, revealed that PB application maintained the phytochemical efficiency, thereby enhancing the submergence tolerance [27,54]. Our transcriptomic analysis further revealed that 10 DEGs were significantly enriched in the “Photosynthesis–antenna proteins” pathway, with most of them upregulated in PBT compared with the control or GAT. The results suggested that maintaining a relatively high expression level of antenna protein genes is an important way for PB to enhance rice submergence tolerance. 

Reserves of non-structural carbohydrates [55], including starch and soluble sugars, are key for rice plant recovery after submergence [4]. As previously reported, we detected obvious retention of NSC in the PB-treated plants. Our transcriptomic analysis further revealed that a number of important sucrose and starch metabolism regulator genes were differentially expressed in GAT, PBT, and the control. For example, *OsCyt-inv1* (*cytosol invertase 1*) (*LOC_Os02g34560*), encoding an alkaline/neutral invertase, is responsible for the conversion of sucrose into glucose and fructose. Mutation of *OsCyt-inv1* resulted in short roots and accumulation of sucrose, and exogenous supply of glucose could repair root growth defects in the mutant, suggesting its positive roles in sucrose metabolism [56]. Our transcriptomic data showed that GAT significantly upregulated *OsCyt-inv1* expression, while PBT maintained *OsCyt-inv1* expression at a similar level as the control, thus suppressing sucrose metabolism under submergence. Meanwhile, we also found a significantly higher expression level of alpha-amylase gene *OsAmy3D* (*LOC_Os08g36910*) in the GAT. *OsAmy3D* is known as a key regulator of starch degradation for the energy for rice anaerobic growth [57]. The higher level of *OsAmy3D* in GAT may explain the faster consumption of starch, and PBT suppressed *OsAmy3D* expression to save more energy for recovering. In addition to the gene mentioned above, other starch or sucrose metabolism-related genes, such as *RSR1* (*Rice Starch Regulator1*) (*LOC_Os05g03040*) [58] and *OsSPP* (*sucrose-6F-phosphate phosphohydrolase*) (*LOC_Os01g27880*) [59], were differentially regulated, indicating their involvement in the submergence response.

In summary, we identified 3936 DEGs among GA-treated, PB-treated, and untreated seedlings under submergence stress. Physiological assays and DEG functional annotation analysis suggested that PB significantly enhances rice seedling survival by promoting a higher level of chlorophyll content and alcohol dehydrogenase activity, and decelerating the consumption of non-structural carbohydrates compared with the control and GA-treated samples. Application of PB drove the expression of several submergence-related genes, such as *CIPK15*, *MPK3*, *SD-1*, and *OsCyt-inv1*, to activate the corresponding pathways to enhance submergence tolerance. Hence, molecular manipulation of these key genes using submergence-inducible promoters may have the potential to genetically improve rice submergence tolerance.

## 4. Materials and Methods

### 4.1. Plant Materials and Growth Conditions

This study was conducted in the greenhouse of the China National Rice Research Institute in natural conditions during May to June of 2015. Rice submergence-intolerant variety IR64 (*Oryza sativa* ssp. *indica*) plants were grown in plastic pots (38 cm in length, 28 cm in width, and 12 cm in height) filled with 2.0 kg mixture of dry paddy soil and special seedling substrate (NPK content ≥ 5%, organic material ≥ 15%) in a 4:1 ratio; a 24-15-15 (N-P_2_O_5_-K_2_O) fertilizer was applied at 0.8 g per pot rate once before seeding. Thirty IR64 pre-germination seeds were sown in each pot. Irrigation was applied as needed to prevent any visible drought stress during seedling growth. Plants were thinned to 20 seedlings per pot 10 days after sowing. 

### 4.2. Treatments and Experimental Design

An experiment based on randomized complete block design with 10 pots as 10 replications was used to test the effect of paclobutrazol (PB) (CAS:76738-62-0, Sigma-Aldrich, St. Louis, MO, USA) and gibberellic acid (GA_3_) (CAS:77-06-5, Sigma-Aldrich, St. Louis, MO, USA) on submergence stress responses of young rice seedlings. PB and GA_3_ stock solutions were made by pre-dissolving 300 and 150 mg powders in 2 mL 95% ethanol and then diluting them to 200 mg·L^−1^ and 100 mg·L^−1^ by water, respectively. Twenty days after germination (DAG), seedlings were sprayed with 25 mL of 200 mg·L^−1^ PB, 100 mg·L^−1^ GA_3_, or methanol solvent (0.1% final concentration) as control two days prior to the submergence. Plants were completely submerged and water depth was maintained at 100 cm for 0, 4, 8, 12, and 16 days in concrete tanks (180 cm in length, 120 cm in width, and 100 cm in height). The tank water was not changed during the treatment.

### 4.3. Sampling and Measurements

Plant height of 10 seedlings was measured just before and after submergence. Seedlings of three pots were harvested after submergence, and leaves were immediately frozen in liquid nitrogen and stored at −80 °C for the alcohol dehydrogenase (ADH) activity assay. ADH activity was quantified as described by previous study [60]. ADH enzyme activities in extracts were measured sepectrophotometrically at A_340_ at 25 °C for 2 min, as described by Ellis and Setter (1999) [61]. Each measurement was repeated three times.

After submergence, four pots of plant samples were milled for measurement of soluble sugar and starch content. Samples of 100 mg of ground tissue powders were extracted three times for 20 min at 80 °C with 10 mL of 90% (*v*/*v*) ethanol. After centrifugation at 3000× *g* for 10 min, the supernatant was collected and placed in a new tube, and the ultimate volume was set to 50 mL. One milliliter of supernatant was collected and transferred into a glass tube. Total soluble sugar content was quantified by the anthrone method with glucose used as the standard [62]. The ethanol-insoluble fraction was dried in an oven at 80 °C for starch extraction. Briefly, 2 mL of distilled water was added to the tubes containing the dried residue. The tubes were put in a boiling water bath for 15 min, and added with 2 mL 9.2 N HClO_4_ for 15 min. The suspension is then made up to 10 mL and centrifuged. Supernatant was then collected, and the residues were extracted again with 2 mL 4.6 N HClO_4_ for 15 min, and then made up to 10 mL with distilled water. The collected supernatants were merged to a final volume of 50 mL. Starch content in the supernatant was determined according to a previous report [62]. Each measurement had four replications. 

Seedlings from another three pots were allowed to recover for seven days after submergence, and the survival rate was recorded as: survival% = (with green leaves after seven days of submergence/total seedlings) × 100. Chlorophyll content was measured by a SPAD meter (Konica Minolta, Japan) before and just after submergence and 7 d later. The SPAD value of top, completely developed leaves of 10 seedlings was measured for each treatment. 

### 4.4. Construction of the mRNA Libraries and High-Throughput Sequencing

Total RNA was extracted from leaves of seedlings using Trizol (Invitrogen, Carlsbad, CA, USA) according to the manufacturer’s protocols. The total RNA quantity and purity were analyzed by Bioanalyzer 2100 and RNA 6000 Nano Labchip Kit (Agilent, Carlsbad, CA, USA) with RIN number > 7. 0.5 µg of total RNA representing a specific adipose type was subjected to isolate Poly (A) mRNA with poly-T oligo attached magnetic beads (Invitrogen). The mRNA is fragmented into small pieces using a divalent cation under elevated temperature after purification, then the cleaved RNA fragments were reverse-transcribed to create the final cDNA library in accordance with the protocol for the mRNA-Seq sample preparation kit (Illumina, San Diego, CA, USA); the average insert size for the paired-end libraries was 300 bp (±50 bp). The paired-end sequencing was performed on an Illumina Hiseq2000/2500 sequencer (LC Sciences, Houston, TX, USA). Nine RNA libraries consisted of three control libraries, three PB-treated libraries, and three GA-treated libraries. 

### 4.5. RNA-Seq Analysis

Prior to assembly, low-quality reads, including reads containing sequencing adaptors or sequencing primer or nucleotide with q quality score < 20, were removed. The raw sequence data have been submitted to the NCBI Short Read Archive with accession number GSE104035. The obtained clean reads were mapped to the rice reference genome in rice genome annotation project database: (ftp://ftp.plantbiology.msu.edu/pub/data/Eukaryotic_Projects/o_sativa/annotation_dbs/pseudomolecules/version_7.0/all.dir/) using the Tophat package. The aligned read files were processed by Cufflinks, which uses the normalized RNA-seq fragment counts to measure the relative abundances of the transcripts. The unit of measurement is Fragment per Kilobase of exon per Million fragments mapped (FPKM). Cufflink was used to de novo assemble the transcriptome. Only the comparisons with a “*q* value” less than 0.01 and status marked as “OK” in the Cuffdiff output were regarded as showing differential expression.

### 4.6. Quantitative RT-PCR

Quantitative RT-PCR was performed as described by Hou et al. (2017) [63]. Briefly, four micrograms of total RNA was applied for reverse transcription using first strand cDNA synthesis Kit (Toyobo, Shanghai, China). Real-time quantitative RT-PCR was performed in a total reaction volume of 20 µL, which includes 10 µL THUNDERBIRD SYBR^®^ qPCR Mix (Toyobo, Shanghai, China), 1 µL cDNA, 1 µL primers, and 8 µL water, on a Bio-Rad CFX96 real-time PCR detection system (Bio-Rad, Hercules, CA, USA). The relative expression level was calculated by the 2^−ΔΔ*C*t^ method. The experiment was performed in three biological replicates. Ubiquitin gene was used as an internal control. The sequence of primers is listed in Table S4. 

## Supplementary Materials

Supplementary materials can be found at www.mdpi.com/1422-0067/18/10/2225/s1.
