# Supplementary figures and images for: Transcriptomic Analysis of Gibberellin- and Paclobutrazol-Treated Rice Seedlings under Submergence
Source: Int J Mol Sci. 2017 Oct 24;18(10):2225. doi: 10.3390/ijms18102225 (PMC5666904; doi:10.3390/ijms18102225)

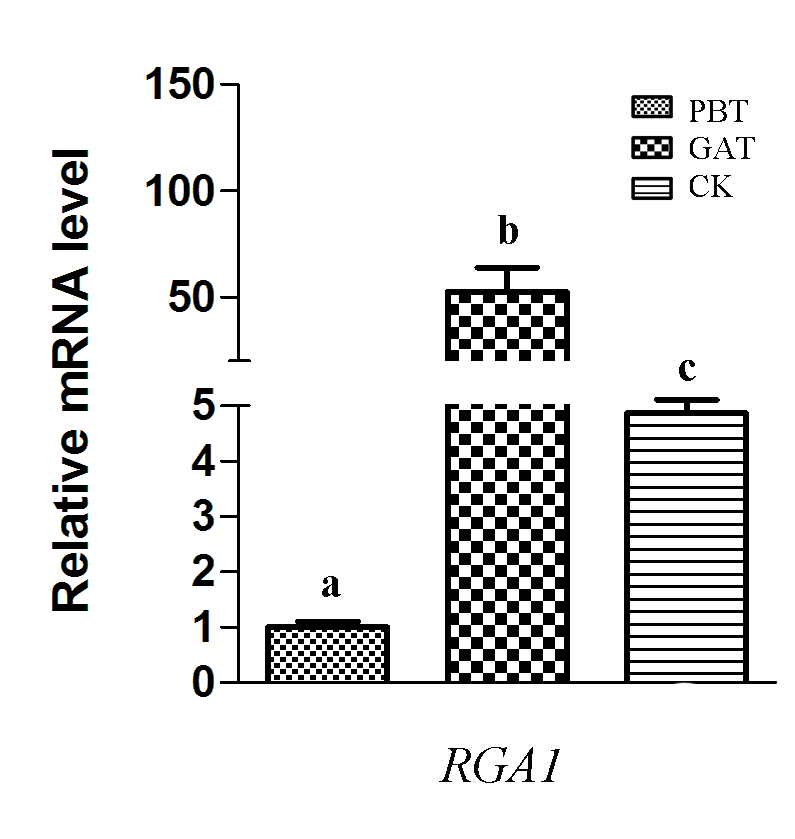

Supplement: Supplementary file 1 [file ijms-18-02225-s001.zip › supplementary files/Figure S1.tif]
